# Genetic and environmental determinants of violence risk in psychotic disorders: a multivariate quantitative genetic study of 1.8 million Swedish twins and siblings

**DOI:** 10.1038/mp.2015.184

**Published:** 2015-12-15

**Authors:** A Sariaslan, H Larsson, S Fazel

**Affiliations:** 1 Department of Psychiatry, Warneford Hospital, University of Oxford, Oxford, UK; 2 Department of Medical Epidemiology and Biostatistics, Karolinska Institutet, Stockholm, Sweden

## Abstract

Patients diagnosed with psychotic disorders (for example, schizophrenia and bipolar disorder) have elevated risks of committing violent acts, particularly if they are comorbid with substance misuse. Despite recent insights from quantitative and molecular genetic studies demonstrating considerable pleiotropy in the genetic architecture of these phenotypes, there is currently a lack of large-scale studies that have specifically examined the aetiological links between psychotic disorders and violence. Using a sample of all Swedish individuals born between 1958 and 1989 (*n=*3 332 101), we identified a total of 923 259 twin-sibling pairs. Patients were identified using the National Patient Register using validated algorithms based on International Classification of Diseases (ICD) 8–10. Univariate quantitative genetic models revealed that all phenotypes (schizophrenia, bipolar disorder, substance misuse, and violent crime) were highly heritable (*h*^2^=53–71%). Multivariate models further revealed that schizophrenia was a stronger predictor of violence (*r*=0.32; 95% confidence interval: 0.30–0.33) than bipolar disorder (*r*=0.23; 0.21–0.25), and large proportions (51–67%) of these phenotypic correlations were explained by genetic factors shared between each disorder, substance misuse, and violence. Importantly, we found that genetic influences that were unrelated to substance misuse explained approximately a fifth (21% 20–22%) of the correlation with violent criminality in bipolar disorder but none of the same correlation in schizophrenia (*P*_bipolar disorder_<0.001; *P*_schizophrenia_=0.55). These findings highlight the problems of not disentangling common and unique sources of covariance across genetically similar phenotypes as the latter sources may include aetiologically important clues. Clinically, these findings underline the importance of assessing risk of different phenotypes together and integrating interventions for psychiatric disorders, substance misuse, and violence.

## INTRODUCTION

Systematic reviews have found that individuals who are diagnosed with psychotic disorders (for example, schizophrenia and bipolar disorder) have an increased likelihood of committing violent acts when compared with general population controls.^1, 2^ Nationwide registry studies have further observed that such associations persist even when patients are compared with their unaffected siblings,^2, 3, 4^ which suggests that the associations may be consistent with a causal inference.^5^ Importantly, these studies add to a broader literature that attributes a large share of the violence risk increase to a subgroup of patients who have or are currently engaging in substance misuse.^6, 7^ Such comorbidity contributes to poor impulse control and non-adherence to either psychological or pharmacological treatments, which have been identified as important risk factors for violence in patients suffering from psychotic disorders.^8, 9, 10^ Nevertheless, it currently remains largely unclear what aetiological factors (for example, genes and environments) link psychotic disorders, with and without comorbid substance misuse, to acts of violence.

Twin and family studies have consistently demonstrated that psychotic disorders, substance misuse and violent crime aggregate in families, in large part due to genetic influences.^11, 12, 13^ The genetic architecture of psychotic disorders is characterized by considerable pleiotropy, as evidenced by large-scale quantitative genetic studies, suggesting that approximately half of the genetic influences increasing the liabilities of developing schizophrenia and bipolar disorder may be shared.^11^ These findings were recently replicated with molecular genetic data, where the genetic correlation between schizophrenia and bipolar disorder, based on single-nucleotide polymorphisms, was estimated to be 0.68 (95% confidence interval: 0.64–0.72).^14^ Further, a 2015 study of nearly 3.5 million Swedish adults indicated that a general genetic factor accounted for between 10 and 36% of the phenotypic variance in six common psychiatric disorders, including schizophrenia and bipolar disorder, as well as substance misuse and violent crime.^15^ Notably, the same study extracted two additional genetic factors that also contributed meaningfully to the aetiologies of these phenotypes. A more detailed analysis is therefore needed to reveal the extent to which the specific sources of covariance between psychotic disorders and violent crime are aetiologically distinct from substance misuse. To the best of our knowledge, there are currently no quantitative genetic studies that have specifically examined the aetiological overlap between psychotic disorders and violent crime.

In the present study, we have used nationwide Swedish data for around 1.8 million individuals born between 1958 and 1989 to address this uncertainty. We aimed to provide estimates of the relative contributions of genes and environments to the overlap between psychotic disorders and violent crime. Given the high rates of comorbid substance misuse in these patient groups, we additionally sought to stratify the aetiological determinants to those that were either shared with substance misuse and those that were unique to each disorder. The clarification of these aetiological links is important to inform psychiatric genetic studies and may assist in developing improved risk assessment and management in these disorders.

## Materials and methods

### Participants

Statistics Sweden maintains all nationwide Swedish longitudinal registries with routinely gathered governmental data. The linkage of individual-level data between different nationwide registers is possible via a unique 10-digit civic registration number assigned to all Swedish residents at birth or upon immigration.^16^ To ensure confidentiality, we were granted access to de-identified data after approval from the Regional Research Ethics Committee at Karolinska Institutet (2009/939–31/5). Informed consent was therefore not required.

We used the Swedish Total Population Register to identify all individuals born in Sweden between 1958 and 1989 and linked their data with the Swedish Twin Registry^17^ and the Multi-Generation Register to identify all monozygotic (MZ) twins, dizygotic (DZ) and non-twin full-siblings. The Cause of Death Register provided mortality dates between 1961 and 2009. Individuals who were born before 1961 were therefore only included if they were registered to be alive at the end of 1960 in the national census register. The Migration Register supplied emigration dates throughout the studied period. We identified individuals with hospital discharges using the National Patient Register that encompasses data on psychiatric inpatient care since 1973 (International Classification of Diseases (ICD) 8−10) and specialist outpatient care since 2001 (ICD 10). A complete list of the ICD-codes used to define our measures is available in Supplementary Table 1. The National Crime Register supplied detailed information on all criminal convictions in lower general court in Sweden since 1973. The practice of plea-bargaining is prohibited in Sweden and conviction data include all individuals who received custodial or non-custodial sentences as well as those who were transferred to forensic psychiatric hospitals. Convictions additionally include cases where the prosecutor decided to caution or fine the individual. The legal age of responsibility is 15 years in Sweden; hence, we were unable to identify offenders younger than this age. We obtained data on socio-demographic factors from the national census registers, available every 5th year between 1970 and 1990 and annually thereafter until 2009.

The sample included all individuals born in Sweden between 1958 and 1989, who had not died or migrated before the age of 15 years (*n=*3 232 010). We identified all MZ twins (*n=*12 588), DZ twins (*n=*27 148) and non-twin full-siblings (*n=*2 369 775) in this sample. In families with multiple offspring, we selected the oldest two siblings born within 5 years from one another to reduce bias because of violation of the equal environment assumption for siblings in our analyses. The final sample included 923 259 twin-sibling pairs (6294 MZ pairs, 13 574 DZ pairs and 903 391 non-twin full-sibling pairs).

### Measures

Individuals who had been hospitalised with any diagnosis of schizophrenia on at least two separate occasions, irrespective of any psychiatric comorbidity, were defined as having schizophrenia. Validation studies examining single-episode hospital admissions for schizophrenia in the National Patient Register have reported satisfactory validity^3, 18, 19^ but this strict definition is commonly used in the literature to minimise the risk of identifying false-positive cases.^11^ We identified individuals with bipolar disorder using a validated algorithm,^20^ which requires a diagnosis of bipolar disorder on at least two separate occasions and excludes a diagnosis of schizophrenia.

In line with other work,^21, 22^ we defined violent crime a conviction for homicide, assault, robbery, threats and violence against an officer, gross violation of a person's integrity, recurrent intimate partner violence directed at a woman,^23^ unlawful threats, unlawful coercion, kidnapping, illegal confinement, arson, intimidation or sexual offences (rape, indecent assault, indecent exposure or child molestation, but excluding prostitution, hiring of prostitutes or possession of child pornography). We used an omnibus measure of substance misuse that included alcohol and drug-related convictions (crimes against the Narcotic Drugs Act (SFS 1968:64) and driving under the influence of alcohol and/or illicit substances) as well as hospital admissions for either alcohol or drug-related disorders.

Family disposable income, defined as the standardized net sum of earnings and benefits, averaged across both biological parents, at the end of the year that the offspring had reached 15 years of age, was used as a proxy measure for the material living standard during adolescence. If this information was unavailable, we used data from the previous year or until it became available. Previous studies have shown that psychiatric disorders, substance misuse and violent criminality are all non-linearly distributed across socioeconomic strata in Sweden, with the heaviest concentrations being observed in the most deprived groups.^21, 24, 25^ We therefore decided to measure income as a binary variable (lowest decile vs top nine deciles) in the analyses. Approximately 0.2% (*n=*5694) individuals lacked data on this measure and were consequently excluded in the descriptive analyses. Single status was defined as never having been married either at first diagnosis or at age 30 years (for control subjects). Immigrant background was defined as having at least one biological parent who was born outside of Sweden.

### Analytical approach

First, descriptive statistics were generated to examine the differences in socio-demographic characteristics and comorbidities across the three psychiatric disorders. From the population sample, we identified healthy control subjects (*n=*2 425 703) by excluding individuals who had ever been hospitalised for any diagnosis of a psychiatric disorder.

Using the twin and sibling data, we subsequently specified a series of univariate quantitative genetic structural equation models for each of the four phenotypes (for example, schizophrenia, bipolar disorder, substance misuse, and violent crime). This approach allowed us to decompose the phenotypic variation of each phenotype into three distinct sources of influence: (a) additive genetic influences, (b) shared environmental influences (for example, environmental factors that make twins and siblings phenotypically more similar to one another, such as family socioeconomic status, parental separation and parental premature mortality) and (c) unique environmental influences (for example, environmental factors that make twins and siblings phenotypically different from one another, such as exposure to different peer groups and experiencing traumatic events in adulthood).^26^ Similar to the classical twin model, we assumed that MZ twins shared all of their co-segregating genes, whereas DZ twins and non-twin full-siblings shared half of their co-segregating genes.^5^ We further assumed that all twins and siblings shared their childhood family environments but that they where completely uncorrelated in regards to their unique environmental influences.^27^ To accommodate the binary nature of the phenotypes under examination, we specified the models to adopt the liability-threshold approach, which assumes that the liability to endorse a given phenotype is approximately normally distributed.^26^ Sex and birth year were included in the models as measured covariates to adjust the thresholds for such group differences.

We subsequently tested for the stability of our findings against confounding and model misspecification. We re-fitted the models after removing individuals with an immigrant background (*n=*54 341); and those who had either died or migrated after their 15th birthday (*n=*220 072).

Last, we fitted two separate multivariate quantitative genetic models using Cholesky decomposition, which enabled us to estimate the phenotypic correlations with violent crime for each psychiatric disorder and decompose the correlations into the relative contributions of genetic and environmental influences that were either shared with substance misuse or specific to each disorder.^28^ All of the quantitative genetic models were fitted in R/OpenMx 1.3.^29^

## Results

In the population sample, we identified 10 265 (0.3%) and 12 627 (0.4%) individuals who met criteria for schizophrenia and bipolar disorder, respectively, as well as 2 425 703 healthy control subjects (Table 1). The mean age at first diagnosis was slighter lower for schizophrenia patients (mean age: 28.1 years, s.e.m.=7.7) than for bipolar disorder patients (mean age: 31.2 years, s.e.m.=8.9). There were less women among schizophrenia patients (35%) than bipolar disorder (62%). Although both patient groups were found to have poorer social and medical functioning outcomes throughout their life-course (for example, being raised in a low-income family, single status at the time of first admission, and premature mortality) as well as lifetime comorbidities (for example, substance misuse and violent crime) when compared with the controls, these were substantially higher in schizophrenia patients than in patients diagnosed with bipolar disorder. We observed, for instance, that nearly one in four (23%) schizophrenia patients had ever been convicted of a violent crime, whereas the equivalent prevalence was 11% in patients diagnosed with bipolar disorder and 3% in controls.

The quantitative genetic models were fitted on a sample consisting of 1 846 518 individuals, of which 12 588 were MZ twins, 27 148 DZ twins and 1 806 782 non-twin full-siblings (Table 2). There were in total 923 259 twin-sibling pairs and the prevalence rates for schizophrenia (0.3–0.4%), bipolar disorder (0.4–0.5%) were stable across all groups of twins and siblings. Similarly, we found that the distribution of socio-demographic factors and comorbidities were stable across the groups of twins and siblings within each disorder.

**Figure 1:**
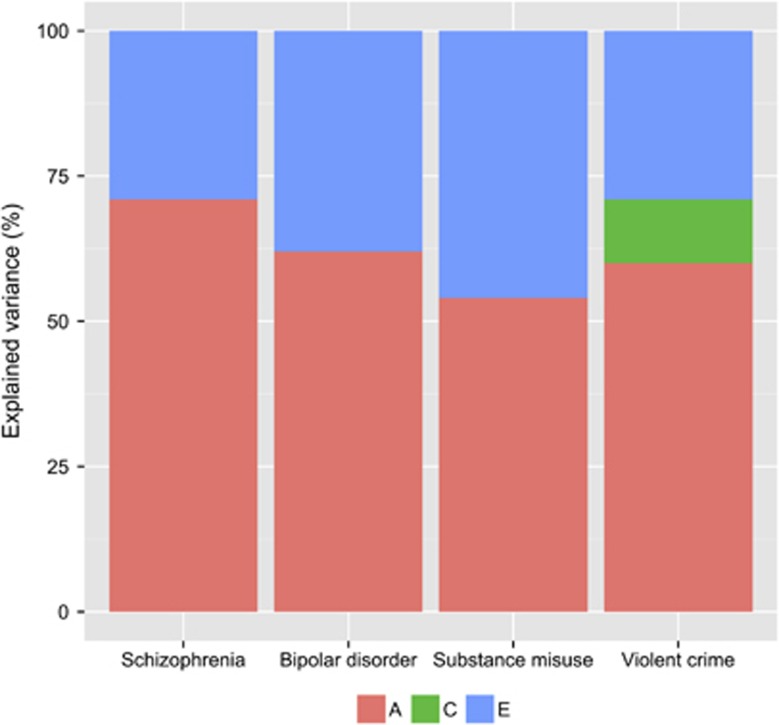
Univariate quantitative genetic models decomposing the variance of schizophrenia, bipolar disorder, substance misuse, and violent crime into additive genetic (A), shared environmental (C) and unique environmental influences (E).

Figure 1 demonstrates that the heritability (for example, the proportion of variance in a trait that is attributed to additive genetic factors; *h*^2^) was substantial for schizophrenia (*h*^2^=71% 95% confidence interval: 65–77%), bipolar disorder (*h*^2^=62% 57–67%) and substance misuse (*h*^2^=53% 51–54%). The remaining variance was entirely explained by unique environmental influences. In contrast, we found that the familial aggregation of violent crime was explained by both additive genetic (*h*^2^=54% 47–61%) and shared environmental influences (14% 7–21%). These estimates were stable across sub-samples excluding individuals with immigrant background as well as individuals who had either died or migrated during follow-up (Supplementary Table 2).

**Figure 2:**
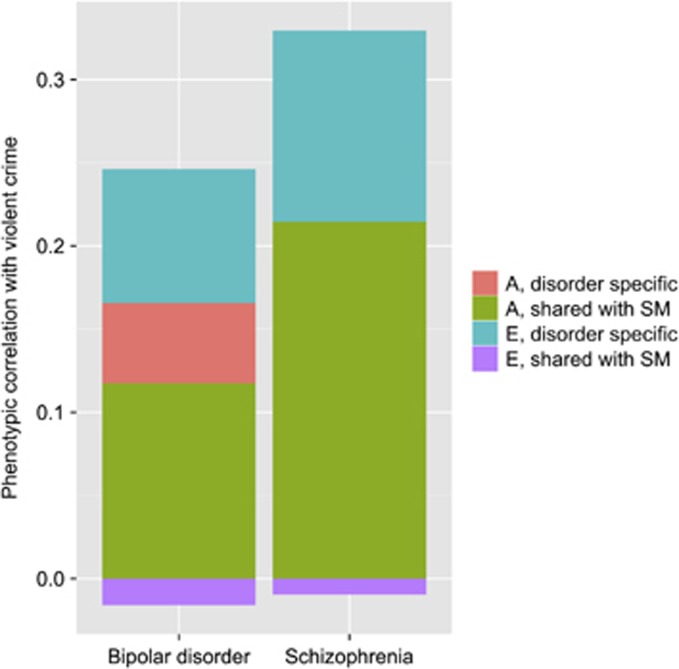
Multivariate quantitative genetic models decomposing the phenotypic correlation between psychotic disorders (schizophrenia and bipolar disorder) and violent crime into additive genetic (A) and unique environmental influences (E) that are shared with substance misuse (SM) and those that are specific to each disorder.

The multivariate quantitative genetic models (Figure 2) confirmed that schizophrenia patients were more likely to be convicted of a violent crime (*r*=0.32; 0.30–0.33) than patients diagnosed with bipolar disorder (*r*=0.23; 0.21–0.25). Large proportions (51–67%) of the bivariate correlations between the psychotic disorders and violent crime were explained by additive genetic influences shared with substance misuse. In the case of schizophrenia, we found no evidence for additive genetic influences that were unrelated to substance misuse (for example, disorder-specific genetic influences; *P=*0.55). In contrast, however, such influences accounted for 21% (20–22%) of the correlation between bipolar disorder and violent crime. Unique environmental influences shared between the psychotic disorders and substance misuse were inversely associated with violence risk, which implies that once genetic factors were accounted for, unique environmental factors that increased the risk of both psychotic disorders and substance misuse tended to decrease the risk of violent crime. Although statistically significant, these influenced merely accounted for 3–7% of the correlations. In contrast, disorder-specific unique environmental influences were positively associated with the risk of violent crime convictions and explained between 35 and 36% of the correlations. Complete correlation matrices are presented in Supplementary Tables 3 and 4.

## Discussion

We report a population-based quantitative genetic study examining the aetiological links between psychotic disorders and violent crime using a sample of ~1.8 million Swedish individuals born between 1958 and 1989. Consistent with previous population-based research,^11, 12, 13^ we found substantial heritability estimates for schizophrenia, bipolar disorder, substance misuse and violent crime (*h*^2^=53–71%). Schizophrenia was a stronger predictor of violent crime (*r*=0.32) than bipolar disorder (*r*=0.23). Consistent with previous quantitative and molecular genetic studies demonstrating important pleiotropic effects between the examined phenotypes,^13, 14, 15^ we found that 67% of the correlation between schizophrenia and violent crime, and 51% of the correlation between bipolar disorder and violent crime was attributed to additive genetic influences that were shared between the psychotic disorders, substance misuse, and violent crime. This suggests that the increased risk for violence in these patient groups could largely be attributed to the same genetic factors that simultaneously increased their liabilities to substance misuse and to be diagnosed with the psychotic disorders in the first place. However, we additionally observed a novel finding in that the additive genetic influences that were unrelated to substance misuse explained approximately a fifth (21%) of the correlation with violent criminality in bipolar disorder but none of the same correlation in schizophrenia. In other words, we found support for the existence of disorder-specific genetic effects linking bipolar disorder (but not schizophrenia) to increased violence risk. This implies that aetiological models involving psychotic disorders should not only focus on genetic and environmental factors that are shared between the disorders but also on those factors that are unique to each disorder.

Explanations for this bipolar-specific genetic effect could include the following points: Clinical studies have demonstrated that impulsivity and risk-taking behaviours are stronger in bipolar disorder than in schizophrenia^30^ and the rates of aggression and violence tend to be more pronounced in patients diagnosed with bipolar disorder who exhibit higher rates of impulsivity.^31^ Second, it has also been established that the risk of aggression in bipolar disorder increases substantially during acute manic episodes,^31, 32^ which are characterised by elevated levels of irritability, impatience and impaired insight.^33^ Genetically informative longitudinal clinical studies are therefore warranted to further explore the ways in which symptoms of mania interplay with substance misuse to generate increased levels of aggression and violence.

We were unable to tell, based on our methodological approach, what specific genes and environmental factors that influenced the risk for violent crime. The available genome-wide association studies on adulthood antisocial behaviours have thus far been too small and heterogeneous to yield any robust findings and comprehensive meta-analyses have failed to identify a single significant polymorphism associated with aggression.^34, 35^ Similarly, only a handful of single-nucleotide polymorphisms have been found to be individually associated with different substance misuse phenotypes but a recent study adopting the genome-wide complex trait analysis approach found that the combined influence of all single-nucleotide polymorphisms explained between 25% to 36% of the variance in different substance misuse phenotypes.^36^ Data on five common psychiatric disorders from the PGC were recently re-examined using multivariate genome-wide complex trait analysis models and the authors concluded that the genetic risk predictions had improved substantially with the addition of multiple genetically similar phenotypes.^37^ The presented findings indicate that the additional inclusion of substance misuse and aggression/violence phenotypes into multivariate molecular genetic studies will likely contribute to greater improvements of statistical power and accuracy. However, the findings also highlight problems with not disentangling common and unique sources of covariance across genetically similar phenotypes as the latter sources may include aetiologically important clues.

In line with previous Swedish nationwide studies that studied these associations using differentially exposed full-siblings,^2, 3, 38^ we found moderately sized unique environmental influences, which are analogous to within-family estimates.^5^ This implies that the increased risk of violence in individuals diagnosed with the psychotic disorders was, to a moderate extent (35–36%), attributed to their individual differences, or ‘unique environmental influences', that could not be explained by their familial characteristics. Unique environmental risks that were shared between the psychiatric disorders and substance misuse, on the other hand, tended to decrease the risk of violence but the effect sizes were marginal (3–7%). This replication is an important confirmation of the conclusions drawn by the previous studies as our research design is not as sensitive to misclassification bias and residual confounding due to non-shared genetic risks between non-twin siblings.^5, 39^

In terms of specific unique environmental influences, it has been demonstrated in a number of genetically informative general Swedish population studies that socioeconomic deprivation and urban residence are merely correlates and not causes of either violent crime, substance misuse or psychiatric morbidity,^21, 24, 25^ whereas the association between peer deviance and drug abuse,^40^ and associations between sustaining a traumatic brain injury and subsequent violent crime^41^ and suicide^42^ appear to be consistent with a causal inference. These findings highlight the importance of accounting for unobserved familial confounders in future studies of putative modifiable environmental risk factors on violence, especially in psychiatric patient groups, as the pleiotropic genetic influences are substantial across all of these traits.

One clinical implication of the findings is that, because of the reported strong overlap between these phenotypes, the treatment of the mental illness and comorbid substance misuse should be integrated more fully in mental health services, which in turn may have benefits for patient safety and public health by reducing violence risk. Consistent with the recent UK NICE guideline on treatments for patients diagnosed with psychotic disorders and comorbid substance misuse,^43^ these findings also highlight the importance of considering psycho-education provided to the patients and their families as a routine and repeated intervention about the risks of substance misuse. Moreover, the prevention of violent behaviour needs to be considered in all patients, particularly in those with first episodes as contact with the criminal justice system may worsen prognosis. Such preventative strategies could include avoidance of antisocial peers^40^ and other interventions to reduce the risks of being victimized and other risk factors.

A number of limitations need to be noted. First, the findings related explicitly to the twins and siblings included in the sample. However, the prevalence rates of the phenotypes in the latter sub-sample did not differ materially from equivalent rates in the population sample (Tables 1 and 2) and the univariate models replicated the estimates of related studies that compared full and half-siblings.^11, 12, 13^ Second, although the presented estimates (for example, phenotypic correlations and the relative contributions of genetic and environmental influences) are valid for the entire population, we cannot exclude the possibility that they are moderated by gender. We were nevertheless unable to explore such moderation effects because the stratification into gender-specific twin sub-samples resulted in some bivariate tables containing cells with zero observations, which prevented the reliable estimation of the tetrachoric correlations. Pooling data from different nations with national registers could be explored to address important question. Further work could also examine different crime outcomes, and in particular, sexual crimes as there is now some preliminary evidence that heritability estimates may differ for certain subgroups (for example, contact vs non-contact sexual offences).^44^

Third, we could not reliably ascertain their temporal ordering of the phenotypes because they represent severe end points to complex developmental processes. Future aetiological studies should therefore benefit from combining nationwide registry data with detailed longitudinal clinical and subclinical measures of psychotic experiences, substance misuse, aggression and antisocial behaviours. Fourth, we relied on validated definitions of schizophrenia and bipolar disorder that were formulated to ensure a high degree of specificity. Complementary sensitivity analyses using wider definitions of the disorders (for example, at least one episode instead of two episodes) were not materially different with the presented findings (Supplementary Table 5).

Last, the specification of the quantitative genetic models presented here assumes the absence of both non-random mating and dominance genetic influences. Furthermore, they assume that the shared environments and prevalence rates of the phenotypes are approximately equal across the groups of twins and non-twin full-siblings. Non-random mating has been established for many of the phenotypes^12, 45^ but a violation of this assumption will only contribute to a downward bias of the additive genetic influences.^27^ Simulation models comparing models with and without dominance genetic influences rarely observe that the inclusion of such effects changes the overall share of the variance attributed to genetic influences.^46^ We decided against modelling for such influences to keep the complex models more parsimonious. The equal environments assumption is violated if the shared environmental correlation in MZ twins exceeds that of DZ twins and non-twin full-siblings, which will cause an artificial increase of the heritability estimate.^26^ However, the impact of this assumption is minimal at best as a recent simulation study demonstrated that the presence of such bias increases heritability estimates by a maximum of 5%.^27^

## Conclusions

We found that patients who were diagnosed with psychotic disorders had elevated risks of committing violent offences largely due to genetic influences that simultaneously increased their liabilities to develop their psychiatric morbidity, to engage in substance misuse and also commit violent crimes. Genetic influences that were unique to bipolar disorder explained approximately a fifth of the increased risk of violent crime while equivalent genetic influences in schizophrenia did not contribute to such risk increases. Future research efforts should be directed towards integrating violence-related phenotypes into psychiatric genetic studies and to identify potentially causal unique environmental predictors of violence in these patient groups. Clinically, these findings illustrate the importance of risk assessments that consider substance misuse comorbidity, integrated treatments for multiple adverse risks and strong collaborations between criminal justice, substance misuse and mental health services.

## Figures and Tables

**Table 1:** Descriptive data for individuals diagnosed with psychotic disorders and controls in the population sample (*n=*3 232 010)

	*Schizophrenia (*n*=10 265; 0.3%)*	*Bipolar disorder (*n*=12 627; 0.4%)*	*Unaffected controls (*n*=2 425 703; 75.1%)*
*Socio-demographic factors*			
Age at 1st diagnosis, years (s.e.m.)	28.1 (7.7)	31.2 (8.9)	NA
Female, *n* (%)	3600 (35.0)	8507 (62.2)	1 094 902 (45.1)
Immigrant background, *n* (%)	397 (3.9)	311 (2.3)	37 262 (1.5)
Income in the lowest decile, *n* (%)	1651 (16.1)	1745 (12.8)	226 482 (9.4)
Single, *n* (%)	7158 (91.8)	8564 (67.8)	1 699 422 (77.1)
Died during follow-up, *n* (%)	979 (9.5)	484 (3.5)	30 569 (1.3)
Emigrated during follow-up, *n* (%)	286 (2.8)	507 (3.7)	146 654 (6.1)
			
*Lifetime comorbidities*			
Substance misuse, *n* (%)	3004 (29.3)	3652 (26.7)	77 281 (3.2)
Violent crime, *n* (%)	2383 (23.2)	1492 (10.9)	73 879 (3.1)

Abbreviation: NA, not applicable.

**Table 2:** Descriptive data for the distribution of individuals across non-twin full-siblings (*n=*2 369 775), DZ twins (*n=*27 148) and MZ twins (*n=*12 588) nested within the patient groups in the sample used for the quantitative genetic models

	*Schizophrenia (*n*=5549; 0.3%)*			*Bipolar disorder (*n*=7509; 0.4%)*		
	*Non-twin full-siblings*	*DZ twins*	*MZ twins*	*Non-twin full-siblings*	*DZ twins*	*MZ twins*
*Socio-demographic factors*						
Age at 1st diagnosis, years (s.e.m.)	28.1 (7.6)	29.1 (7.6)	29.7 (8.8)	31.4 (8.6)	32.1 (9.0)	32.2 (9.3)
Female, *n* (%)	1906 (35.3)	40 (36.7)	13 (39.4)	4,503 (61.6)	91 (63.2)	39 (68.4)
Immigrant background, *n* (%)	52 (1.0)	2 (1.8)	3 (9.1)	18 (0.2)	1 (0.7)	0
Income in the lowest decile, *n* (%)	741 (13.7)	17 (15.6)	6 (18.2)	766 (10.5)	24 (16.7)	4 (7.0)
Single, *n* (%)	3795 (91.8)	77 (90.6)	27 (90.0)	4,515 (67.0)	87 (64.4)	33 (66.0)
Died during follow-up, *n* (%)	476 (8.8)	8 (7.3)	0	247 (3.4)	5 (3.5)	2 (3.5)
Emigrated during follow-up, *n* (%)	163 (3.0)	2 (1.8)	0	286 (3.9)	5 (3.5)	0
						
*Lifetime comorbidities*						
Substance misuse, *n* (%)	1514 (28.0)	28 (25.7)	7 (21.2)	1,919 (26.3)	37 (25.7)	13 (22.8)
Violent crime, *n* (%)	1173 (21.7)	19 (17.4)	9 (27.3)	764 (10.5)	14 (9.7)	7 (12.3)
Number of patients	5407	109	33	7308	144	57
Prevalence (95% CI)	0.3% (0.3–0.3%)	0.4% (0.3–0.5%)	0.3% (0.2–0.4%)	0.4% (0.4–0.4%)	0.5% (0.4–0.6%)	0.5% (0.3–0.6%)

Abbreviations: CI, confidence interval; DZ, dizygotic; MZ, monozygotic.

95% CIs for the prevalence rates were calculated using the binomial exact method.
