# Supplementary material for: Genetic and environmental determinants of violence risk in psychotic disorders: a multivariate quantitative genetic study of 1.8 million Swedish twins and siblings
Source: Mol Psychiatry. 2015 Dec 15;21(9):1251–6. doi: 10.1038/mp.2015.184 (PMC4842006; doi:10.1038/mp.2015.184)
Supplement: Supplementary Table 1 [file mp2015184x1.docx]

**eTable 1 ICD diagnostic codes**

|  | **ICD-8** | **ICD-9** | **ICD-10** |
| --- | --- | --- | --- |
| Schizophrenia | 295 | 295 | F20 |
| Bipolar disorder | 296.0-296.3 [excl. 296.2], 296.8, 296.9 | 296A-296E [excl. 296B], 296W, 296X | F30-F31 |
| Alcohol and drug-related disorders | 291-292, 303-304 | 291-292, 303-304, 305A, 305X | F10-F12, F14-16, F19 |
