# Supplementary material for: Genetic and environmental determinants of violence risk in psychotic disorders: a multivariate quantitative genetic study of 1.8 million Swedish twins and siblings
Source: Mol Psychiatry. 2015 Dec 15;21(9):1251–6. doi: 10.1038/mp.2015.184 (PMC4842006; doi:10.1038/mp.2015.184)
Supplement: Supplementary Table 2 [file mp2015184x2.docx]

**eTable 2 Sensitivity tests: Comparing estimates across sub-samples excluding individuals with an immigrant background and those who either emigrated or died during follow-up**

|  | **Additive genetic influences** | **Shared environmental influences** | **Unique environmental influences** |
| --- | --- | --- | --- |
| ***Schizophrenia*** |  |  |  |
| Main sample | 71% [65%; 77%] | 0% [0%; 0%] | 29% [23%; 35%] |
| Excluding individuals with an immigrant background | 71% [65%; 77%] | 0% [0%; 0%] | 29% [24%; 35%] |
| Excluding those who either emigrated/died during follow-up | 73% [67%; 80%] | 0% [0%; 0%] | 27% [20%; 33%] |
|  |  |  |  |
| ***Bipolar disorder*** |  |  |  |
| Main sample | 62% [57%; 67%] | 0% [0%; 0%] | 38% [33%; 43%] |
| Excluding individuals with an immigrant background | 71% [65%; 77%] | 0% [0%; 0%] | 29% [23%; 35%] |
| Excluding those who either emigrated/died during follow-up | 64% [58%; 69%] | 0% [0%; 0%] | 36% [31%; 42%] |
|  |  |  |  |
| ***Substance misuse*** |  |  |  |
| Main sample | 53% [51%; 54%] | 0% [0%; 0%] | 47% [46%; 49%] |
| Excluding individuals with an immigrant background | 52% [51%; 54%] | 0% [0%; 0%] | 48% [46%; 49%] |
| Excluding those who either emigrated/died during follow-up | 51% [50%; 52%] | 0% [0%; 0%] | 49% [48%; 50%] |
|  |  |  |  |
| ***Violent criminality*** |  |  |  |
| Main sample | 54% [47%; 61%] | 14% [7%; 21%] | 32% [28%; 35%] |
| Excluding individuals with an immigrant background | 58% [45%; 71%] | 12% [5%; 19%] | 30% [23%; 36%] |
| Excluding those who either emigrated/died during follow-up | 59% [46%; 73%] | 11% [4%; 17%] | 30% [24%; 37%] |
