# Supplementary material for: Genetic and environmental determinants of violence risk in psychotic disorders: a multivariate quantitative genetic study of 1.8 million Swedish twins and siblings
Source: Mol Psychiatry. 2015 Dec 15;21(9):1251–6. doi: 10.1038/mp.2015.184 (PMC4842006; doi:10.1038/mp.2015.184)
Supplement: Supplementary Table 4 [file mp2015184x4.docx]

**eTable 4 Within-trait and between-trait correlations for twin and sibling pairs for substance misuse, bipolar disorder and violent crime**

| **MZ twins** | | | | | | |
| --- | --- | --- | --- | --- | --- | --- |
|  | **Twin I** | | | **Twin II** | | |
|  | Substance misuse | Bipolar disorder | Violent crime | Substance misuse | Bipolar disorder | Violent crime |
| **Twin I** |  |  |  |  |  |  |
| Substance misuse | … | 0.35  [0.17; 0.53] | 0.54  [0.46; 0.63] | 0.56  [0.48; 0.63] | 0.11  [-0.14; 0.36] | 0.47  [0.38; 0.55] |
| Bipolar disorder | … | … | 0.14  [-0.11; 0.40] | 0.33  [0.14; 0.51] | 0.79  [0.67; 0.91] | 0.27  [0.07; 0.48] |
| Violent crime | … | … | … | 0.46  [0.37; 0.55] | 0.30  [0.09; 0.50] | 0.75  [0.70; 0.81] |
| **Twin II** |  |  |  |  |  |  |
| Substance misuse | … | … | … | … | 0.38  [0.20; 0.55] | 0.49  [0.41; 0.57] |
| Bipolar disorder | … | … | … | … | … | 0.33  [0.14; 0.53] |
| Violent crime | … | … | … | … | … | … |
|  | | | | | | |
| **DZ twins** | | | | | | |
|  | **Twin I** | | | **Twin II** | | |
|  | Substance misuse | Bipolar disorder | Violent crime | Substance misuse | Bipolar disorder | Violent crime |
| **Twin I** |  |  |  |  |  |  |
| Substance misuse | … | 0.39  [0.28; 0.49] | 0.54  [0.49; 0.59] | 0.28  [0.22; 0.34] | 0.03  [-0.14; 0.20] | 0.14  [0.06; 0.22] |
| Bipolar disorder | … | … | 0.22  [0.08; 0.35] | 0.10  [-0.05; 0.25] | 0.31  [0.11; 0.52] | 0.08  [-0.09; 0.25] |
| Violent crime | … | … | … | 0.17  [0.10; 0.25] | 0.08  [-0.10; 0.25] | 0.28  [0.20; 0.35] |
| **Twin II** |  |  |  |  |  |  |
| Substance misuse | … | … | … | … | 0.35  [0.24; 0.47] | 0.48  [0.43; 0.54] |
| Bipolar disorder | … | … | … | … | … | 0.15  [-0.02; 0.31] |
| Violent crime | … | … | … | … | … | … |
|  | | | | | | |
| **Non-twin full-siblings** | | | | | | |
|  | **Sibling I** | | | **Sibling II** | | |
|  | Substance misuse | Bipolar disorder | Violent crime | Substance misuse | Bipolar disorder | Violent crime |
| **Sibling I** |  |  |  |  |  |  |
| Substance misuse | … | 0.37  [0.35; 0.38] | 0.53  [0.52; 0.53] | 0.25  [0.25; 0.26] | 0.08  [0.06; 0.10] | 0.25  [0.24; 0.25] |
| Bipolar disorder | … | … | 0.18  [0.16; 0.20] | 0.10  [0.08; 0.12] | 0.30  [0.27; 0.33] | 0.07  [0.05; 0.09] |
| Violent crime | … | … | … | 0.25  [0.24; 0.26] | 0.09  [0.07; 0.11] | 0.37  [0.36; 0.38] |
| **Sibling II** |  |  |  |  |  |  |
| Substance misuse | … | … | … | … | 0.35  [0.34; 0.37] | 0.52  [0.51; 0.52] |
| Bipolar disorder | … | … | … | … | … | 0.18  [0.16; 0.20] |
| Violent crime | … | … | … | … | … | … |
