# Supplementary material for: Genetic and environmental determinants of violence risk in psychotic disorders: a multivariate quantitative genetic study of 1.8 million Swedish twins and siblings
Source: Mol Psychiatry. 2015 Dec 15;21(9):1251–6. doi: 10.1038/mp.2015.184 (PMC4842006; doi:10.1038/mp.2015.184)
Supplement: Supplementary Table 5 [file mp2015184x5.docx]

**eTable 5 Sensitivity tests: Comparing alternative definitions of psychotic disorders.**

|  | **Phenotypic correlation with violence (rPh)** | **Proportion of rPh attributed to:** | | | |
| --- | --- | --- | --- | --- | --- |
|  |  | **Additive genetic influences shared with substance misuse** | **Disorder-specific additive genetic influences** | **Unique environmental influences shared with substance misuse** | **Disorder-specific unique environmental influences** |
| **Schizophrenia** |  |  |  |  |  |
| At least one episode | 0.32 [0.32; 0.33] | 0.62 [0.61; 0.63] | -0.06 [-0.08; -0.04] | -0.02 [-0.02; -0.02] | 0.46 [0.45; 0.46] |
| At least two episodes | 0.32 [0.30; 0.33] | 0.67 [0.65; 0.68] | 0.00 [-0.01; 0.00] | -0.03 [-0.03; -0.02] | 0.36 [0.35; 0.37] |
|  |  |  |  |  |  |
| **Bipolar disorder** |  |  |  |  |  |
| At least one episode | 0.25 [0.25; 0.26] | 0.50 [0.48; 0.51] | 0.09 [0.07; 0.11] | -0.06 [-0.06; -0.05] | 0.47 [0.45; 0.49] |
| At least two episodes | 0.23 [0.21; 0.25] | 0.51 [0.47; 0.54] | 0.21 [0.20; 0.22] | -0.07 [-0.07; -0.06] | 0.35 [0.34; 0.35] |
